# Bevacizumab confers significant improvements in survival for ovarian cancer patients with low miR-25 expression and high miR-142 expression

**DOI:** 10.1186/s13048-021-00915-9

**Published:** 2021-11-22

**Authors:** Jun Li, Huiran Yue, Wenzhi Li, Guohua Zhu, Tingting Zhu, Ruifang Chen, Xin Lu

**Affiliations:** 1 Present Address: Department of Gynecology, Obstetrics and Gynecology Hospital, Fudan University, No.419, Fangxie Road, Shanghai, 200011 People’s Republic of China; 2 Shanghai Key Laboratory of Female Reproductive Endocrine Related Diseases, Shanghai, 200011 China

**Keywords:** Ovarian cancer, Lymphovascular space invasion, MicroRNA, Bevacizumab, Prognosis

## Abstract

**Background:**

Lymphovascular space invasion (LVSI) is the first step of hematogenous metastasis. Exploration of the differential miRNA expression profiles between LVSI-positive and LVSI-negative ovarian cancer tissues may help to identify key miRNAs involved in the hematogenous metastasis of ovarian cancer. This study is aimed to identify microRNAs (miRNAs) that are differentially expressed between LVSI-positive and LVSI-negative ovarian cancer tissues, followed by exploring their association with bevacizumab response in ovarian cancer patients.

**Methods:**

The Cancer Genome Altas (TGGA) dataset was used to identify the differentially expressed miRNAs between LVSI-positive and LVSI-negative ovarian cancer tissues. The prognostic value of the differentially expressed miRNAs was determined using GSE140082 dataset.

**Results:**

We showed that miR-25 and miR-142 were differentially expressed between LVSI-positive and LVSI-negative ovarian cancer tumors. Kaplan-Meier analysis indicated that high miR-25 expression was associated with increased progression free survival (PFS) and extended overall survival (OS). Moreover, patients with low miR-25 expression benefited significantly from bevacizumab treatment in terms of PFS. A similar trend was observed in terms of OS though without reaching statistical significance. In contrast, no significant survival benefits from bevacizumab were observed in patients with high miR-25 expression in terms of PFS and OS. There was no significant correlation between miR-142 expression and PFS. In contrast, high miR-142 expression was associated with reduced OS. Moreover, patients with high miR-142 expression benefited significantly from bevacizumab treatment in terms of PFS and OS. However, bevacizumab treatment conferred no significant improvements in both PFS and OS in patients with low miR-142 expression. The nomogram for PFS indicated that miR-25 expression had a larger contribution to PFS than debulking status and bevacizumab treatment. And the nomogram for OS illustrated both miR-25 expression and miR-142 expression as sharing a larger contribution to OS than bevacizumab treatment and debulking status.

**Conclusion:**

In conclusion, miR-25 expression correlates with a better PFS and OS in ovarian cancer. Patients with low miR-25 expression and high miR-142 expression could benefit from bevacizumab treatment significantly.

## Background

Ovarian cancer is the most lethal gynecological malignancy, and most of the cases are not diagnosed until advance stages [1]. Patients with this disease suffer for poor prognosis, mainly due to the widespread peritoneal and distant metastases. Exploration of the mechanisms involved in ovarian cancer metastasis may help to improve the clinical outcome of these patients.

For many years, ovarian cancer has been widely believed to spread via a passive mechanism [2]. Namely, ovarian cancer cells are shed from the primary sites and implant at the surface of the peritoneal organs aided by the peritoneal circulation [2]. However, a predilection for omental implantation has promoted scientists to consider an alternate mechanism of ovarian cancer metastasis to the omentum that hangs down from the stomach [3, 4]. In addition, the presence of retroperitoneal, distant, and submesothelial metastases also raise the possibility of alternate routes of metastasis [5]. Moreover, scientists have identified circulating tumor cells (CTCs) in the blood samples of ovarian cancer patients, indicating a blood-borne route of ovarian cancer metastasis [6]. Indeed, the work by Pradeep and colleagues have suggested that ovarian cancer is able to metastasize to the omentum hematogenously [4]. Although scientists have demonstrated the importance of hematogenous metastasis in ovarian cancer, the underlying mechanisms remain to be elucidated. In the metastasis of cancer cells through hematogenous route, lymphovascular space invasion (LVSI) is the first step. The cancer cells first invade into and then transit in blood or lymphatic vessels. Subsequently, these cells undergo extravasation and establish secondary tumors at the metastatic sites. Thus, exploration of the differential miRNA expression profiles between LVSI-positive and LVSI-negative ovarian cancer tissues may help to identify key miRNAs involved in the hematogenous metastasis of ovarian cancer.

In this study, we tried to identify miRNAs that were differentially expressed between LVSI-positive and LVSI-negative ovarian cancer tissues, and subsequently explored the association of LVSI-related miRNAs with bevacizumab response in ovarian cancer patients.

## Materials and methods

### Patient cohort

LVSI information and miRNA profiling were available in 191 ovarian cancer patients in the TCGA dataset. For these patients, we downloaded the level 3 miRNA expression data. Differential expressed genes between LVSI-positive and LVSI-negative tumors are identified using the R package Limma [7]. Fold change> 1.25 or < 0.80 with adjusted *P* value< 0.05 were considered to be statistically significant.

GSE140082 dataset was used to explore the prognostic value of LVSI-associated miRNA in ovarian cancer. Namely, univariate and multivariate analysis were used to explore the association of miR-25 and miR-142 with progression free survival (PFS) and overall survival (OS) in patients with ovarian cancer. Bevacizumab response were also evaluated according to the expression level of miR-25 and miR-142.

### Statistical analysis

Limma R package was used to identify the differentially expressed miRNAs between LVSI-positive and LVSI-negative ovarian cancer tissues. “Low” and “high” was defined according to the gene expression level. The cutoff values for miR-25 and miR-142 were determined using survminer R package. Continuous data between two groups and categorical data were compared using two independent samples t-test and chi-square or Fisher’s exact test where appropriate, respectively. Univariable and multivariable analyses were used to identify factors associated with PFS and OS. Details of the development of the prognostic nomograms were described in our previous published paper [8]. The statistical analyses were performed using IBM SPSS Statistics (version 22.0) and R (version 3.5.2). Two-sided *P* value < 0.05 was considered statistically significant.

## Results

### Identification of miRNAs associated with LVSI status in ovarian cancer patients

To identify LVSI-associated miRNAs in ovarian cancer, we analyzed the miRNA expression profiling of 135 LVSI-positive and 56 LVSI-negative ovarian cancer tissues from TCGA database. Totally, 16 miRNAs and 18 miRNAs were downregulated (fold change< 0.80) and upregulated (fold change> 1.25) in LVSI-positive group versus LVSI-negative group with a *P* value< 0.05, respectively (Table 1). Among these differentially expressed miRNAs, only two miRNAs’ (miR-25 and miR-142-5p) adjusted *P* value were less than 0.05 (Table 1).

**Table 1:** MiRNAs that were differentially expressed between LVSI-positive and LVSI-negative ovarian cancer tissues

Downregulated miRNA	LVSI(+)/LVSI(−) Fold change	*P* value	Adjusted *P* value	Upregulated miRNA	LVSI(+)/LVSI(−) Fold change	*P* value	Adjusted *P* value
hsa-miR-25	0.778	< 0.001	0.025	hsa-miR-142-5p	1.651	< 0.001	0.025
hsa-miR-96	0.614	0.002	0.143	hsa-miR-22	1.516	< 0.001	0.063
hsa-miR-183	0.700	0.004	0.161	hsa-miR-142-3p	1.600	< 0.001	0.065
hsa-miR-514	0.625	0.008	0.180	hsa-miR-21	1.272	< 0.001	0.065
hsa-miR-218	0.716	0.007	0.180	hsa-let-7b	1.334	0.001	0.082
hsa-miR-335	0.770	0.008	0.180	hsa-miR-21^*^	1.426	0.002	0.143
hsa-miR-509-5p	0.742	0.013	0.204	hsa-miR-199a-5p	1.444	0.005	0.180
hsa-miR-375	0.793	0.012	0.204	hsa-miR-150	1.388	0.004	0.180
hsa-miR-26b	0.758	0.015	0.222	hsa-miR-483-5p	1.324	0.008	0.180
hsa-miR-200a	0.747	0.016	0.225	hsa-miR-202	1.277	0.005	0.180
hsa-miR-125b	0.785	0.018	0.229	hsa-miR-199b-3p	1.373	0.012	0.204
hsa-miR-509-3p	0.719	0.026	0.276	hsa-miR-214	1.360	0.012	0.204
hsa-let-7e	0.793	0.029	0.284	hsa-miR-638	1.338	0.014	0.212
hsa-miR-507	0.782	0.039	0.312	hsa-miR-1225-5p	1.345	0.018	0.229
hsa-miR-99a	0.790	0.040	0.315	hsa-miR-188-5p	1.269	0.017	0.229
hsa-miR-509-3-5p	0.730	0.045	0.323	hsa-miR-222	1.265	0.024	0.267
				hsa-miR-144	1.355	0.028	0.284
				hsa-miR-223	1.330	0.030	0.286

*is part of the name of a specific miRNA

### The impact of miR-25 and miR-142 expression on PFS and OS in ovarian cancer

**Fig. 1:**
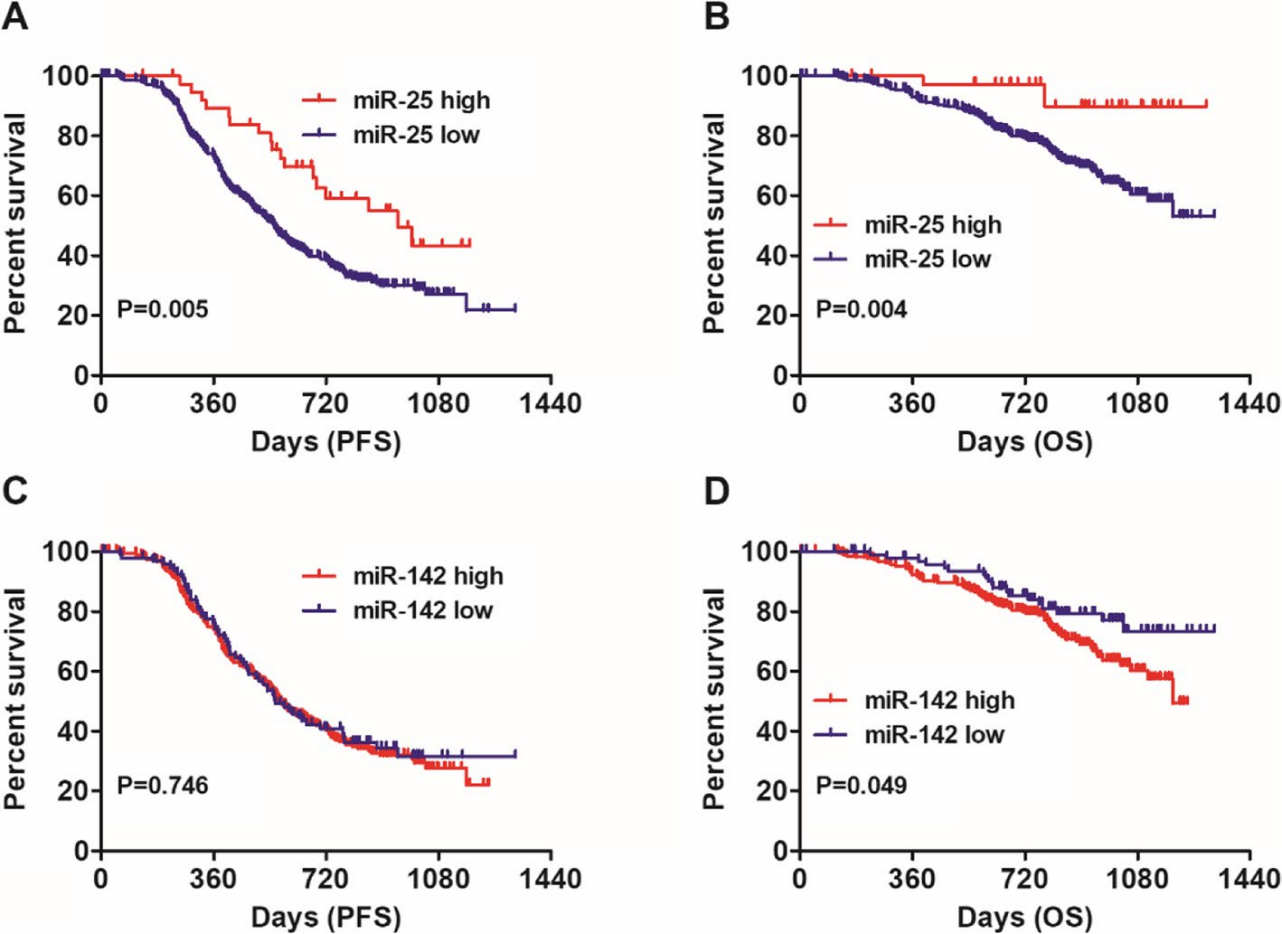
The association of miR-25 and miR-142 expression with OS. **A** MiR-25 was associated with increased PFS (HR: 0.59, 95%CI: 0.40–0.85, Log-rank *P* = 0.005) in GSE140082 dataset; **B** MiR-25 was associated with extended OS (HR: 0.42, 95%CI: 0.23–0.76, Log-rank *P* = 0.004) in GSE140082 dataset; **C** There was no significant correlation between miR-142 expression and PFS (HR: 1.05, 95%CI: 0.78–1.41, Log-rank *P* = 0.746) in GSE140082 dataset; **D** MiR-142 was associated with reduced OS (HR: 1.56, 95%CI: 1.00–2.43, Log-rank *P* = 0.049) in GSE140082 dataset

Then, we explored the associations of miR-25 and miR-142 expression with the survival of ovarian cancer patients. Kaplan Meier analysis indicated that high miR-25 expression was associated with increased PFS (Fig. 1A, HR: 0.59, 95%CI: 0.40–0.85, Log-rank *P* = 0.005) and extended OS (Fig. 1B, HR: 0.42, 95%CI: 0.23–0.76, Log-rank *P* = 0.004). There was no significant correlation between miR-142 expression and PFS (Fig. 1C, HR: 1.05, 95%CI: 0.78–1.41, Log-rank *P* = 0.746). In contrast, high miR-142 expression was associated with reduced OS (Fig. 1D, HR: 1.56, 95%CI: 1.00–2.43, Log-rank *P* = 0.049).

### Bevacizumab response stratified by the expression level of miR-25 and miR-142

**Fig. 2:**
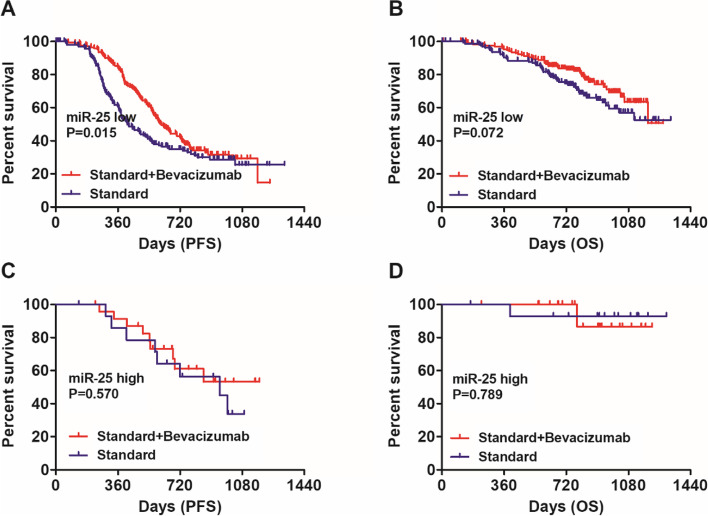
Bevacizumab response stratified by the expression level of miR-25. **A** Bevacizumab conferred significant improvements in PFS for patients with low miR-25 expression (HR: 0.71, 95%CI: 0.54–0.94, Log-rank *P* = 0.015). **B** It seemed that bevacizumab conferred improvements in OS for patients with low miR-25 expression though without reaching statistical significance (HR: 0.69, 95%CI: 0.46–1.03, Log-rank *P* = 0.072). **C** No significant survival benefits from bevacizumab were observed in patients with high miR-25 expression in terms of PFS (HR: 0.75, 95%CI: 0.29–2.00, Log-rank *P* = 0.570). **D** No significant survival benefits from bevacizumab were observed in patients with high miR-25 expression in terms of OS (HR: 1.38, 95%CI: 0.13–14.23, Log-rank *P* = 0.789)

**Fig. 3:**
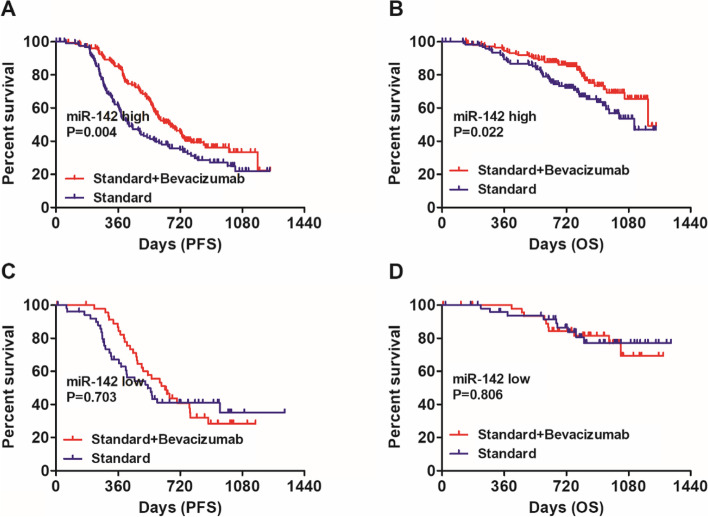
Bevacizumab response stratified by the expression level of miR-142. **A** Bevacizumab conferred significant improvements in PFS for patients with high miR-142 expression (HR: 0.64, 95%CI: 0.47–0.87, Log-rank *P* = 0.004). **B** Bevacizumab conferred significant improvements in OS for patients with high miR-142 expression (HR: 0.59, 95%CI: 0.38–0.93, Log-rank *P* = 0.022). **C** Bevacizumab treatment conferred no significant improvements in PFS (HR: 0.90, 95%CI: 0.54–1.51, Log-rank *P* = 0.703) for patients with low miR-142 expression. **D** Bevacizumab treatment conferred no significant improvements in OS (HR: 1.12, 95%CI: 0.46–2.75, Log-rank *P* = 0.806) for patients with low miR-142 expression

Since LVSI is closely correlated with hematogenous metastasis of cancer cells, we infer that the abundance of LVSI-associated miRNAs may be predictive of response to anti-angiogenesis therapy in ovarian cancer. Thus, we next explored whether molecular subtyping by miR-25 and miR-142 expression analysis could identify patients who would preferentially benefit from bevacizumab treatment. Our results indicated that patients with low miR-25 expression benefited significantly from bevacizumab treatment in terms of PFS (Fig. 2A, HR: 0.71, 95%CI: 0.54–0.94, Log-rank *P* = 0.015). A similar trend was observed in terms of OS (Fig. 2B, HR: 0.69, 95%CI: 0.46–1.03, Log-rank *P* = 0.072) though without reaching statistical significance. In contrast, no significant survival benefits from bevacizumab were observed in patients with high miR-25 expression in terms of PFS (Fig. 2C, HR: 0.75, 95%CI: 0.29–2.00, Log-rank *P* = 0.570) and OS (Fig. 2D, HR: 1.38, 95%CI: 0.13–14.23, Log-rank *P* = 0.789). And patients with high miR-142 expression benefited significantly from bevacizumab treatment in terms of PFS (Fig. 3A, HR: 0.64, 95%CI: 0.47–0.87, Log-rank *P* = 0.004) and OS (Fig. 3B, HR: 0.59, 95%CI: 0.38–0.93, Log-rank *P* = 0.022). However, bevacizumab treatment conferred no significant improvements in both PFS (Fig. 3C, HR: 0.90, 95%CI: 0.54–1.51, Log-rank *P* = 0.703) and OS (Fig. 3D, HR: 1.12, 95%CI: 0.46–2.75, Log-rank *P* = 0.806) in patients with low miR-142 expression.

### Multivariate analysis of the prognostic value of miR-25 and miR-142 in ovarian cancer patients

Next, multivariate analysis further confirmed the positive impact of miR-25 expression on PFS (Table 2) in ovarian cancer patients. However, miR-142 expression was not associated with PFS significantly (Table 2). For OS, the expression of miR-25 had a positive influence on the OS in ovarian cancer patients (Table 3). However, miR-142 expression was associated with reduced OS though without reaching statistical significance (Table 3).

**Table 2:** Univariate and multivariate analysis of the prognostic value of miR-25 and miR-142 in PFS

Variables	Number of patients	Univariate analysis		Multivariate analysis	
		HR (95%CI)	*P* value	HR (95%CI)	*P* value
Age	380	1.015 (1.002–1.027)	0.024	1.012 (0.999–1.025)	0.062
Stage			< 0.001		< 0.001
Early	51	1		1	
Late	329	6.894 (3.400–13.981)		5.861 (2.873–11.954)	
Grade			0.384		/
Low	74	1		/	
High	281	0.871 (0.639–1.188)		/	
NA	25	/		/	
Debulking			0.002		< 0.001
Optimal	290	1		1	
Suboptimal	88	2.115 (1.594–2.807)		1.712 (1.278–2.292)	
Inoperable	2	/			
Treatment			0.009		0.003
Standard	181	1		1	
Standard+Bevacizumab	199	0.711 (0.550–0.918)		0.676 (0.521–0.876)	
miR-25 expression			0.006		0.020
Low	341	1		1	
High	39	0.501 (0.306–0.822)		0.554 (0.338–0.910)	
miR-142 expression			0.747		/
Low	99	1		/	
High	281	1.050 (0.782–1.410)		/

**Table 3:** Univariate and multivariate analysis of the prognostic value of miR-25 and miR-142 in OS

Variables	Number of patients	Univariate analysis		Multivariate analysis	
		HR (95%CI)	*P* value	HR (95%CI)	*P* value
Age	380	1.036 (1.014–1.058)	0.001	1.033 (1.010–1.056)	0.004
Stage			0.003		0.012
Early	51	1		1	
Late	329	5.661 (1.792–17.885)		4.229 (1.324–13.515)	
Grade			0.798		/
Low	74	1		/	
High	281	1.069 (0.644–1.774)		/	
NA	25	/		/	
Debulking			0.002		0.074
Optimal	290	1		1	
Suboptimal	88	1.991 (1.299–3.050)		1.497 (0.962–2.328)	
Inoperable	2	/			
Treatment			0.072		0.087
Standard	181	1		1	
Standard+Bevacizumab	199	0.692 (0.463–1.034)		0.702 (0.468–1.052)	
miR-25 expression			0.009		0.013
Low	341	1		1	
High	39	0.217 (0.069–0.687)		0.233 (0.073–0.738)	
miR-142 expression			0.052		0.091
Low	99	1		1	
High	281	1.648 (0.996–2.727)		1.547 (0.933–2.566)	

### Development of miR-25/miR-142 related nomograms predictive of PFS and OS in ovarian cancer patients

Finally, to quantitatively predict the survival of ovarian cancer patients, we established nomograms incorporating prognostic factors with *P* value< 0.1 indicated by multivariable analysis.

**Fig. 4:**
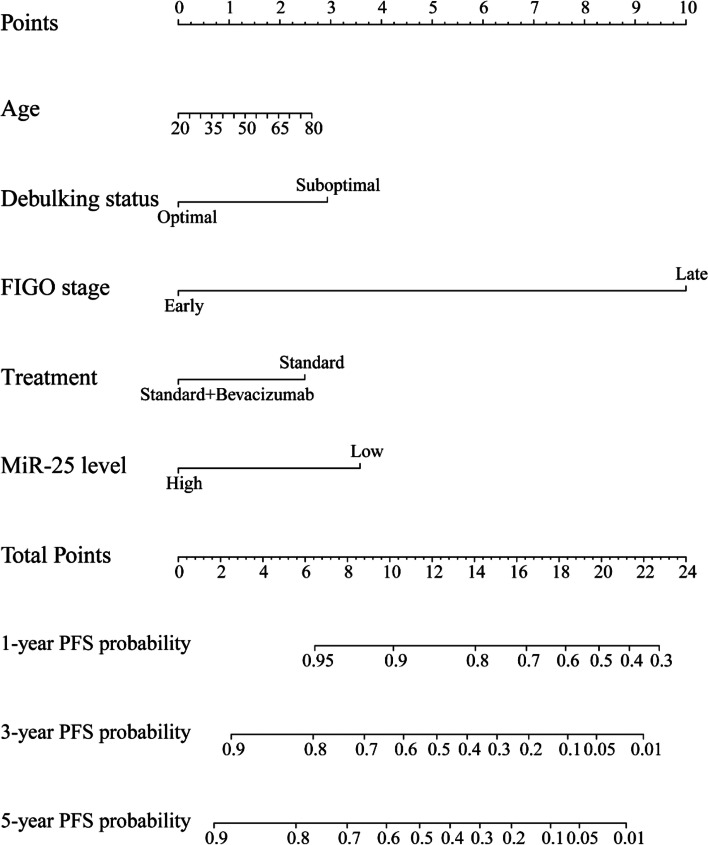
Development of miR-25 related nomograms predictive of PFS in ovarian cancer patients

The nomogram for PFS illustrated FIGO stage as sharing the largest contribution to PFS, followed by miR-25 expression and debulking status (Fig. 2). Notably, miR-25 expression had a larger contribution to PFS than debulking status and bevacizumab treatment (Fig. 4). The C-index for predicting PFS was 0.67 (95%CI, 0.64–0.71).

**Fig. 5:**
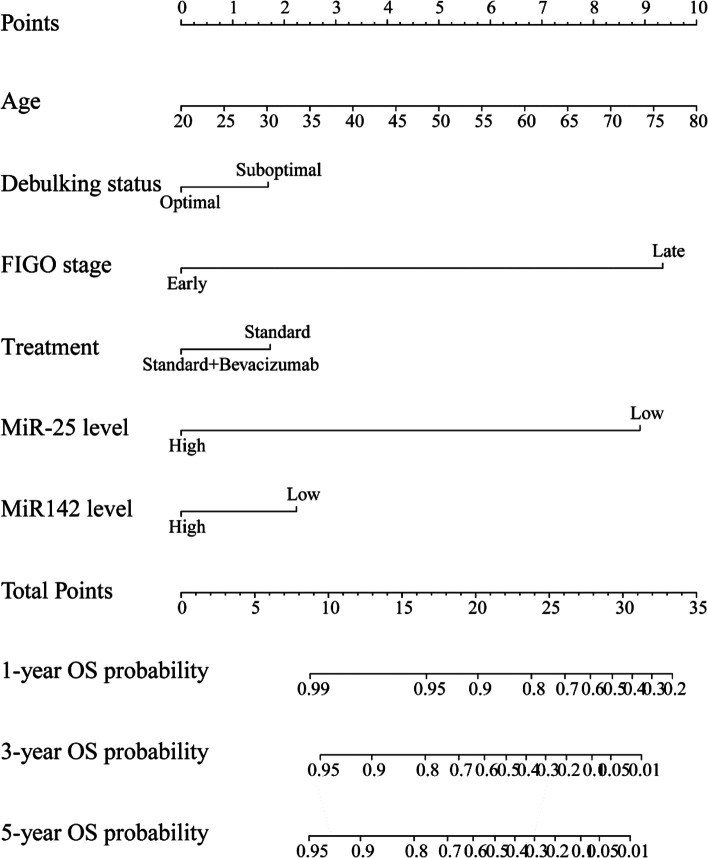
Development of miR-25/miR-142 related nomograms predictive of OS in ovarian cancer patients

The nomogram for OS illustrated age at initial diagnosis as sharing the largest contribution to OS, followed by FIGO stage and miR-25 expression (Fig. 5). Notably, both miR-25 expression and miR-142 expression had a larger contribution to OS than bevacizumab treatment and debulking status (Fig. 5). The C-index for predicting OS was 0.71 (95%CI, 0.66–0.76).

## Discussion

Our previous study indicated that protein-coding genes were differentially expressed between LVSI-positive and LVSI-negative ovarian cancer tissues [9]. Furthermore, a cluster of the LVSI-associated protein-coding genes were closely correlated with survival of ovarian cancer patients [9]. However, the correlation of the non-coding RNA, specifically miRNAs, with LVSI status and survival in ovarian cancer were still elusive. This study was aimed to identify the differentially expressed miRNAs between LVSI-positive and LVSI-negative ovarian cancer tissues, and to explore the prognostic value of these miRNAs.

The major findings of the present study were as follows. First, miR-25 and miR-142 were differentially expressed between LVSI-positive and LVSI-negative ovarian cancer tumors. Second, both low miR-25 expression and high miR-142 expression were associated with bevacizumab response and worse clinical outcomes in ovarian cancer. Third, the nomogram for PFS indicated that miR-25 expression had a larger contribution to PFS than debulking status and bevacizumab treatment. Finally, the nomogram for OS illustrated both miR-25 expression and miR-142 expression as sharing a larger contribution to OS than bevacizumab treatment and debulking status.

MiR-25, a member of the miR-106b ~ 25 cluster, is located within the 13th intron of the minichromosome maintenance complex component 7 (MCM7) gene. The expression of miR-25 was tissue specific. Previous studies showed that miR-25 was upregulated in various cancers, such as breast cancer, endometrial cancer, and so on. However, in prostate cancer and colorectal cancer, the results were contradictory. Some studies showed miR-25 was upregulated in prostate cancer and colorectal cancer. Other studies, however, showed miR-25 was downregulated in prostate cancer and colorectal cancer. In ovarian cancer, Wang et al. showed that miR-25 was upregulated and its expression was positively associated with tumor stage and regional lymph node involvement [10]. Ovarian cancer patients with higher miR-25 expression had a worse survival [10]. However, our study, which had a relatively larger sample size, presented opposite results. The findings from our study were consistent with the findings from the study by Meng et al. [11]. Namely, the serum miR-25 level was significantly decreased in ovarian cancer patients compared with healthy women [11]. Similar results were observed in the study by Langhe et al. [12].

MiR-142, first discovered in chromosome 17 of hematopoietic stem cell, has been demonstrated to be a critical regulator of various biological processes, and dysregulation of miR-142 was associated with various diseases, including cancer. MiR-142 could function as either tumor suppressor or oncogene in a cell-specific manner. For example, miR-142 overexpression could regulate both immune response and tumor microenvironment to initiate a powerful anti-tumor response [13]. In contrast, miR-142 promoted organoid formation by breast cancer stem cells (BCSCs) and enhanced tumor growth initiated by human BCSCs in vivo [14]. In ovarian cancer, miR-142-5p significantly decreased cell proliferation, arrested the cell cycle at the S phase, reduced the ability of colony formation, inhibited cell migration and invasion, and enhances cisplatin-induced apoptosis [15, 16]. Zhang et al. showed that low miR-142 expression was associated with a worse survival [15]. In contrast, our study revealed that low miR-142 expression seemed to be correlated with a better survival. Thus, the prognostic value of miR-142 needs to be further confirmed in future study.

Considering the survival benefit from adding bevacizumab to the frontline therapy in GOG-218 trial and ICON7 trial, bevacizumab was approved as the upfront therapy for ovarian cancer [17, 18]. However, treatment with bevacizumab could be much expensive and could have substantial side effects. Moreover, not all the patients could benefit from the administration of bevacizumab. The survival benefit of bevacizumab was greater in patients at high risk [17, 18]. Thus, it is of great importance to identify the subgroup of patients who may benefit from bevacizumab treatment. In the present study, it was found that ovarian cancer patients with either low miR-25 expression or high miR-142 expression had survival benefit from bevacizumab treatment. Previous studies suggested that miR-25 could downregulate SNAI2 expression by targeting its 3’UTR [19], which may in turn impair endothelial cell activation and angiogenesis [20]. Similarly, miR-142-3p overexpression promoted the production of VEGF-A, which in turn activated angiogenesis [21]. Thus, patients with either low miR-25 expression or high miR-142 expression may have an increased activity of angiogenesis, which may partly explain our observation that bevacizumab confers significant improvements in survival for ovarian cancer patients with either low miR-25 expression or high miR-142 expression. Debulking status is a key determinant for prognostic prediction in ovarian cancer. In this study, the nomogram for OS illustrated both miR-25 expression and miR-142 expression as sharing a larger contribution to OS than debulking status. Combination of FIGO stage, expression of miR-25 and miR-142, debulking status could predict OS more accurately.

## Conclusions

In conclusion, miR-25 expression correlates with a better PFS and OS in ovarian cancer. Patients with low miR-25 expression and high miR-142 expression could benefit from bevacizumab treatment. Further researches are needed to explore the mechanism on the relationship between bevacizumab response and the expression of miR-25 and miR-142.

## Data Availability

Data used in present study are all available in TCGA and GEO databases.
